# Robotic Transanal Total Mesorectal Excision (RTaTME): State of the Art

**DOI:** 10.3390/jpm11060584

**Published:** 2021-06-21

**Authors:** Fabio Rondelli, Alessandro Sanguinetti, Andrea Polistena, Stefano Avenia, Claudio Marcacci, Graziano Ceccarelli, Walter Bugiantella, Michele De Rosa

**Affiliations:** 1 Department of General Surgery and Surgical Specialties, University of Perugia, “S. Maria” Hospital, 05100 Terni, Italy; rondellif@hotmail.com (F.R.); sanguinettiale@gmail.com (A.S.); stefano_avenia@libero.it (S.A.); claudio.marcacci@libero.it (C.M.); 2 Department of General and Laparoscopic Surgery–University Hospital, University of Rome, “Umberto I”, 00161 Rome, Italy; apolis74@yahoo.it; 3 Department of General and Robotic Surgery, “San Giovanni Battista” Hospital, USL Umbria 2, 06034 Foligno, Italy; graziano.ceccarelli@uslumbria2.it (G.C.); walter.bugiantella@uslumbria2.it (W.B.)

**Keywords:** rectal cancer, total mesorectal excision (TME), transanal total mesorectal excision (TaTME), robotic, robotic transanal total mesorectal excision (RTaTme), transanal surgery

## Abstract

Total mesorectal excision (TME) is the gold standard technique for the surgical management of rectal cancer. The transanal approach to the mesorectum was introduced to overcome the technical difficulties related to the distal rectal dissection. Since its inception, interest in transanal mesorectal excision has grown exponentially and it appears that the benefits are maximal in patients with mid-low rectal cancer where anatomical and pathological features represent the greatest challenges. Current evidence demonstrates that this approach is safe and feasible, with oncological and functional outcome comparable to conventional approaches, but with specific complications related to the technique. Robotics might potentially simplify the technical steps of distal rectal dissection, with a shorter learning curve compared to the laparoscopic transanal approach, but with higher costs. The objective of this review is to critically analyze the available literature concerning robotic transanal TME in order to define its role in the management of rectal cancer and to depict future perspectives in this field of research.

## 1. Introduction

Total mesorectal excision (TME) is the standard procedure in the surgical treatment of rectal cancer [1]. In the last two decades this technique has revolutionized the results of rectal cancer surgery, demonstrating how surgical quality has a direct impact on local control and survival [2,3], the circumferential radial margin (CRM) and the integrity of mesorectal envelope being independent predictors of local recurrence [4,5,6]. Similarly, functional results can be considered a direct expression of the quality of surgery and are not always satisfying with conventional surgical techniques for rectal cancer treatment [7,8].

From the first report by Sylla et al. [9], transanal TME (TaTME) aroused great enthusiasm in the colorectal community, showing technical advantages compared to conventional open and laparoscopic approaches, preferentially in clinical scenarios considered “difficult” [10,11].

Indeed, even in the hands of expert surgeons, the rectal resection for distal cancers can be extremely difficult, and this is truer in patients with anterior-located lesions, narrow pelvis, obese, with bulky tumours, or treated with neo-adjuvant chemoradiotherapy, where a challenging distal rectal dissection may increase the risk of incomplete mesorectal excision [12,13].

Exposure of the operative field, rectal dissection, and distal cross-stapling of the rectum can be extremely challenging in these conditions, the latter increasing the risk of anastomotic leakage [14].

TaTME was conceived and developed with the aim of overcoming these limitations, mainly in middle-low tumours. The concept relies on a “bottom-up” (caudal to cephalad) or retrograde dissection technique, in which starting the rectal dissection from the perineum may be advantageous for the surgeon.

A magnified vision in line with pelvic structures, distal control of tumour margin, and improved identification and preservation of nervous structures make dissection from below safe and effective. Moreover, abdominal and pelvic structures and viscera are avoided, as they no longer need to be retracted cephalad for rectal mobilization and exposure.

Several case series have been published in the last eight years, suggesting that taTME is feasible and safe concerning short-term outcomes and quality of the resected specimen, with promising CRM involvement ranging from 0–6% [15].

However, despite this proven safety and feasibility, early reports have shown that TaTME is a challenging technique with a steep learning curve [16,17]. The occurrence of urethral injury, a serious complication directly related to the transanal phase of the operation, [10] and recent evidence of the higher involvement of the distal resection margin (DRM) when compared with a robotic low anterior resection [18] underline how relevant are a deep knowledge of pelvic anatomy and the acquisition of advanced surgical skills.

In this light, the introduction of robotic technology with a stable 3D vision may offer the possibility of performing very complex tasks with ambidextrous movements, decreasing tremor and improved dexterity, thus allowing a better dissection, especially in confined surgical fields [19].

All these advantages would potentially help overcome the steep learning curve related to the complexity of TaTME, making robotic assistance a gold standard for this approach.

## 2. Device and Technique

### 2.1. Robotic Platform

Directly derived from military projects aiming to develop a technology to be used in situations where the expert surgeon is away from the patient, the concept of robotic surgery has become a reality and achieved great success in clinical practice over three decades of scientific and technological progress.

In 1985, the PUMA 560 robotic system was introduced into an operating theatre to orient a needle for a neurosurgical biopsy under computer tomography, providing more accurate and steady guidance compared to a human hand [20]. A transurethral resection of the prostate (TURP) was subsequently performed by Davies et al. using the same technology [21].

Shortly afterwards, the PROBOT was developed by Imperial College, London, and designed specifically to undertake a TURP, while parallel developments led to the introduction of the ROBODOC system (Integrated Surgical Systems, Sacramento, CA, USA), designed to improve the precision of total hip arthroplasties [22]. Currently, ROBODOC is marketed by Curexo Technology Corporation and is the only FDA-approved robot for orthopedic surgery.

At the end of the 20th century, abdominal surgery was revolutionized by the introduction of laparoscopy. In 1994, the Automated Endoscopic System for Optimal Positioning 1000 (AESOP 1000; Computer Motion, Santa Barbara, CA, USA) became the first laparoscopic camera holder to be approved by the FDA and commercialized. It consisted of a table-mounted voice-controlled laparoscopic camera system that adjusted its position following surgeon’s orders, allowing greater image stability and sometimes avoiding the need for an assistant [20].

In 1998, with the introduction of Zeus (Computer Motion, Santa Barbara, CA, USA), the concept of tele-robotics or telepresence was finally applied, with the surgeon sat at a console distant from the robot operating on the patient. With three arms, each independently attached to the surgical table, i.e., one AESOP arm and two surgical arms with four degrees of freedom, a console with a Storz 3D imaging system (Karl Storz Endoscopy, Santa Barbara, CA, USA) and two handles to manipulate the two surgical arms of the telerobot, the Zeus robotic system made a highly relevant mark in the development of cardiac surgery and allowed, in 2001, a transatlantic cholecystectomy with the surgeon operating in New York while the patient was situated physically in Strasbourg, France.

Around the time that ZEUS was being developed, the da Vinci^®^ robot (Intuitive Surgical, Sunnyvale, CA, USA) was introduced. Its console is composed of a computer, a 3D imaging system, and two “masters” that manipulate the arms of the robot and are filtered by the computer to suppress manual tremor. The first da Vinci^®^ robot had three arms, one for the camera and two for surgical instruments, but the latest model has one extra arm for the surgical instruments. Differently to ZEUS, the da Vinci^®^ robot is attached to operative trocars rather than to the surgical table. Hallmark features of this platform are the binocular endoscopic vision which creates a truly 3D experience and specifically designed instruments endowed with Endowrist^®^ technology which, by imitating the human wrist, allow for seven degrees of freedom, 180° of articulation and 540° of rotation [23].

After preliminary experiences such as the undertaking of a cholecystectomy by Himpens in Belgium [24], and a mitral valve replacement by Carpentier et al. [25], in 2000 the da Vinci^®^ obtained FDA approval for general laparoscopic procedures and became the first operative surgical robot in the US.

In 2003 Computer Motion was merged with Intuitive Surgical, the ZEUS and da Vinci^®^ systems were effectively unified and, as a result, further innovations and improvements were focused on the da Vinci^®^ platform, which has subsequently dominated the world of robotic surgery for almost a decade.

From 2014 a more advanced and versatile version of da Vinci^®^, the Xi platform, has become available, offering the opportunity to perform multi-quadrant single-docking procedures with consequently decreased operative time. Augmented-reality software allows the assessment of intestinal perfusion or real-time three-dimensional (3D) anatomical simulation of abdominal structures [26,27].

#### Future Direction: New Robotic Platforms

Since its inception in colorectal surgery, the da Vinci^®^ Surgical System has passed through the evolution of different platforms, and published reports on robotic transanal surgery have shown the feasibility of this approach with currently available systems, but not without remarkable limitations.

The game-changing SP (single-port) da Vinci^®^ Robotic platform has been recently introduced and approved by the FDA for urological procedures, anticipating what is expected to happen soon in colorectal surgery, where pre-clinical or preliminary pilot studies have already demonstrated its feasibility and how promising it might be, mainly in transanal and endoscopic procedures.

Despite the limited clinical use of robotic surgery, driven by economic costs and limited access, the technological advancement in this field of research is under continuous progression.

This list of available surgical robots is non-exhaustive, and many of these projects are confidential at present, with each day bringing newly developed technology.

The Senhance Surgical Robotic System (TransEnterix, Morrisville, NC, USA) obtained clearance by the FDA in October 2017 for gynecological and colorectal procedures [28]. The system includes a “cockpit” that serves as a remote-control station unit, up to four manipulator arms, with their own individual carts, and a HD-3D-technology camera, as well as a haptic feedback system.

The most exciting areas of innovation are single-port and natural orifice surgery, where new robotic platforms with flexible arms and camera are under development.

The Flex^®^ Robotic System and Flex^®^ Colorectal (CR) Drive (MedRobotics, Corp. Raynham, MA, USA), after pilot experiences in oral surgery, proved their feasibility in a cadaveric model and in miscellaneous surgical procedures, gaining FDA approval [29].

Being a semi-robotic platform with no robotic effectors, it does not fully express the potential of current technology, but its special design for transanal endoluminal applications makes it also suitable for more complex tasks such TaTME, as already tested in a preclinical setting.

Other single incision platforms such the Single Port Orifice Robotic Technology–SPORT (Titan Medical Company, Toronto, ON, Canada) or the multi-trocar platforms such as Versius (Cambridge Medical Robotics, Cambridge, UK), Revo-I (model MSR-5000; Meerecompany Inc., Seongnam, Republic of Korea), MiroSurge (Medtronic, Minneapolis, MN, USA), and Medicaroid (Kobe, Japan) are expected to bring further advancement in this field.

Verb Surgical, born in 2015 thanks to the collaboration between Google and Johnson & Johnson, introduced artificial intelligence to robotic systems with the aim of starting a new era of robotic-guided, rather than robot-assisted, surgery. The long-term plan would be to develop a surgery technology fully performed by robots, but this page is yet to be written [30].

### 2.2. Transanal Device

In 1983 TEM (Transanal Endoscopic Microsurgery) was conceived by Professor Gerard Buess who, in cooperation with Richard Wolf, created and developed the platform for endoscopic rectal surgery, with the aim of treating benign lesions of the high and middle rectum not reachable by a conventional transanal approach [31].

Based on this model, Karl Storz developed the TEO (Transanal Endoscopic Operations-Storz, Tuttlingen, Germany), a rigid operative rectoscope which is compatible with many standard laparoscopic instruments and units, and with no need for a dedicated platform.

An evolution and simplification of TEM was introduced in 2009 by Atallah et al. who, by adapting a device already conceived for single-port surgery, created TAMIS (Trans-Anal Minimally Invasive Surgery), a hybrid platform midway between TEM and single-port laparoscopy, using a multiport device placed in the anal canal as an access system, with conventional scope and laparoscopic instruments [32].

To date, two transanal platforms, GelPoint Path (Applied Medical, Rancho Santa Margarita, CA, USA) and SILS Port (Covidien, Mansfield, MA, USA) have gained FDA approval for TAMIS. Clinical studies published so far have demonstrated that both platforms, rigid and non-disposable, e.g, TEM/TEO, and flexible and disposable, e.g., TAMIS, could be equally used for this technique, but TAMIS is the preferred option for surgeons dedicated to transanal surgery because, compared to TEM, it offers a better angle of vision, a soft and less traumatic platform, an economic advantage and an easier set-up [33,34].

A self-designed custom-made transanal access platform, the PAT (Developia-IDIVAL, Santander, Spain), closed with an 80-mm GelPOINT gel cap (Applied Medical, Rancho Santa Margarita, CA, USA) for trocar placement, has been described by Gomez Ruiz et al. [35].

### 2.3. Surgical Technique

Despite the attempt at standardization, with the development of experience and the dissemination of this approach many different technical modifications have been introduced, although the pivotal principle of this procedure remains the same: to provide a complete mobilization of the rectum up to the pelvic floor, regardless of the platform and the device used.

After induction of general anesthesia, patient is catheterized and put in lithotomy position. The procedure is carried out either with two different surgical teams working simultaneously or with a two-step approach, using the same surgical team for both operative phases.

The majority of the experiences reported so far refer to a hybrid procedure that incorporates an abdominal phase to achieve proximal colonic mobilization, inferior mesenteric vessel division and partial rectal dissection, and the transanal part of the operation where the rectal dissection is completed “bottom-up”, joining the surgical plane previously developed.

Abdominal phase: this part of the operation has been described with different variations, either performed simultaneously in a two team approach, or in a sequential model, being the first or the last step in the operation, according to the surgeon’s preference. Similarly, laparoscopic multiport/single-port or robotic-assisted procedures have been described.

Regardless of the approach, operative steps are performed according to the standardized technique of rectal anterior resection with identification and ligation of the inferior mesenteric vessels, full mobilization of the splenic flexure and circumferential incision of the peritoneal reflection to start the rectal dissection posteriorly along the sacral plane, developing it laterally, paying attention not to damage the hypogastric nerves and ureters. Anteriorly, the peritoneal reflection should only be incised, without performing any further dissection manoeuver.

Transanal phase: after pneumoperitoneum release, the transanal phase of the operation can begin using the TAMIS approach with a disposable single-site device, or the reusable platform with rigid rectoscope TEM/TEO. The adoption of a self-designed transanal access port proctoscope PAT (Developia-IDIVAL, Santander, Spain), closed with an 80-mm GelPOINT gel cap (Applied Medical, Rancho Santa Margarita, CA, USA) for trocar placement, has been reported [35].

The placement of a self-fixing anal retractor (Lone Star Medical Products Inc., Houston, TX, USA) may be useful to better expose the anal canal and, once the tumor distance from the anal verge is verified, the procedure can start immediately with an intersphincteric resection or with straight positioning of the anal access system and the creation of a pressure of 8 to 10 mmHg to the pneumopelvis.

In patients with tumors located ≤3 cm from the anal verge, a partial intersphincteric resection can be performed, by circumferential dissection of the mucosa and internal sphincter muscle at least 1 cm below the distal margin of the tumor, developing the plane cranially for 1–2 cm. A purse-string suture is then placed to seal the rectum below the tumor and the transanal access platform is inserted. The robotic trocars are then directly introduced through the GelPOINT Path or, using a trocar in trocar technique, inserted in the gelPOINT path trocars. The da Vinci^®^ system patient cart is positioned near the lower left side of the patient. A 30-degree-angle videoscope, hot shears with monopolar diathermy on the right and a fenestrated grasper (or Maryland grasper) with bipolar cautery on the left are commonly used. An accessory 12-mm trocar is operated by a patient-side assistant, who assists in tissue countertraction or manipulates suction and irrigation modules. If available, an AirSEAL System (Conmed, Utica, NY, USA) and 5-mm or 8-mm valveless trocar can be used for the assistant to stabilize the pneumopelvis. In patients with tumors higher than 3 cm from the anal verge, where an adequate distal resection margin can be obtained without intersphinteric resection, the transanal device is set up directly and the rectum insufflated with CO_2_, establishing the pneumo-rectum.

The rectal mucosa is marked circumferentially with a monopolar hook and a full thickness rectotomy is begun, usually posteriorly, where the plane between presacral fascia and mesorectum is more easily identified, but slightly laterally where the anococcygeal ligament is less easily entered.

The rectal division is performed by opening the different layers of the rectal wall joining the mesorectal plane, which is insufflated to ease the pelvic dissection and RtaTME.

The posterior plane is developed first in the pre-sacral avascular space, along the mesorectal fascia which is kept intact. The anterior plane is approached afterwards, keeping the dissection in front of or behind the Denonvillier’s fascia according to the rectal cancer position in male patients, and dissecting the rectum from the posterior vagina in females. The lateral dissection comes last in order to minimize injuries to the neurovascular structures. Once TME is completed and the peritoneal cavity is accessed, the specimen can be extracted transanally or transabdominally through a suprapubic Pfannenstiel incision using an Alexis wound retractor (Applied Medical Inc., Rancho Santa Margarita, CA, USA). If the transanal route is chosen, the colo-rectal segment is carefully exteriorized and divided, after having checked for the presence of an adequate blood supply.

Different anastomotic techniques have been described according to the surgeon’s experience and the choice should be tailored depending on case specifics and the height of the tumor.

In the case of lower cancers with a short rectal stump, a conventional hand-sewn coloanal anastomosis (lateroterminal when feasible) is performed, while if the rectal stump is long enough to allow a pursestring closure around the anvil of a stapler, a mechanical end-to-end anastomosis should be optioned.

Defunctioning loop ileostomy should be considered to protect low anastomosis, and pelvic drain can be left intra-abdominally.

## 3. Outcomes

Recent reports show similar clinical and oncological results in comparing robotic and laparoscopic trans-abdominal surgical procedures so, at present, no significant benefit of robotic over laparoscopic surgery seems to be detectable, except perhaps in conversion rates [36].

The application of robotics to TaTME appears to be the next logical step in the evolution of minimal access surgery, allowing the technical benefits of an advanced surgical platform, whilst adhering to the principles of NOTES. Despite the literature on RtaTME being in its infancy, this exciting new trend is rising and the results coming from available preclinical or pilot reports are promising in terms of mesorectal integrity, resection margins, number of intraoperatively harvested lymph nodes and conversion rate.

After a preliminary experimental approach in a cadaveric model [37], the first in-human application of robotic technology to TaTME, termed robotic-assisted transanal surgery for TME (RATS-TME), was reported in 2013 [38].

Therefore, after demonstrating the feasibility, Atallah et al. published a case series with the first three human cases performed at a single institution. In all cases, tumors were located in the distal 5 cm of the rectum, no involvement of the distal and circumferential resection margins was detected and no major morbidity or mortality on short-term follow-up were reported [39].

Parallel experience led to the publication of a case of RTaTME performed on a 48-year-old female with a rectal cancer 8 cm from the anal verge, who underwent a sequential laparoscopic and RTaTME using the da Vinci^®^ Si System with the GelPoint Path. The specimen was transanally extracted and an end-to-end stapled anastomosis was fashioned using a circular stapler. The total operative time was 250 min, with an estimated blood loss of 50 mL. No complication was recorded and the postoperative stay was 3 days. The histological report showed a complete mesorectal excision with free distal and circumferential margins [40].

In the single-center preliminary experience of Atallah, from a dataset of 18 robotic miscellaneous transanal procedures, four cases (three male) of distal rectal cancer were treated via a sequential hybrid abdominal laparoscopic and RTaTME (one more patient was added to the previously reported case series.) [39]. The mean operative time was 376 min with an estimated mean blood loss of 200 mL. There was no intra-operative morbidity and the mean postoperative length of stay was 4.3 days. Mesorectal quality was graded as complete or near complete and an R0 resection was performed in all four cases. Concerning morbidity, one wound hematoma, one subsegmental pulmonary embolism (asymptomatic) and recurrent deep vein thrombosis, and a high ileostomy output were recorded. No local or distant recurrences were found in any of the patients after an average 8-month follow-up [41].

Huscher et al. published the results of seven patients (four women) with rectal cancer who underwent a sequential hybrid laparoscopic transabdominal and RTaTME, showing how the combination of robotics and transanal access is feasible and could improve results in rectal cancer surgery. The mean operative time was 165.7 min and no anastomotic leakage was recorded. One patient presented post-operative gastrointestinal bleeding, presumably from the anastomosis, requiring transfusion. The mean hospital stay was 4.8 days. Pathology assessment revealed a complete or near-complete mesorectum and an R0 resection in all cases [42].

Gomez Ruiz et al. performed a pilot study of robotic-assisted laparoscopic transanal proctectomy with TME, enrolling five patients. Mean operative time was 398 ± 88 min with no intraoperative complications. Mean length of hospital stay was 6 ± 1 days. A Clavien II, grade B anastomotic leakage developed in one patient postoperatively. In all cases, specimens showed complete mesorectal excision with negative proximal, distal and circumferential margins. All patients were disease-free at their initial 3-month follow-up [35].

Kuo et al. reported a series of 15 patients (8 males) who underwent a combined sequential single-site plus one port (R-SSPO) robotic transabdominal operation followed by RTaTME performed by a single surgeon adopting the da Vinci^®^ Si surgical system with the da Vinci^®^ Single-Site platform.

Median operative time was 473 min and the estimated blood loss was 33 mL. Conversion to conventional laparoscopy was required in two patients, one due to bleeding during the transanal phase and another due to a left ureteric transection. Reported complications included an intestinal obstruction requiring surgical adhesiolysis and a superficial wound infection. The mean length of hospital stay was 12.2 days. All specimens were reported as complete mesorectum with clear circumferential and distal resection margins [43].

Monsellato et al. reported three consecutive cases (two male) of RTaTME: in two cases a sequential approach with a transanal phase and a subsequent robotic transabdominal operation was performed, and in the third case a simultaneous laparoscopic transabdominal and robotic perineal approach was employed. With a mean operative time of 530 min, no intra-operative or post-operative complications and excellent (Quirke 3 grade) TME quality in all cases, the authors demonstrated that this approach is feasible, safe and with good early post-operative outcomes [44].

In the single-centre experience reported by Hu et al. a total of twenty patients (12 male) underwent RtaTME via a simultaneous two-team approach, with the “abdominal team” working via a laparoscopic single-port technique at ileostomy site, while the “transanal team” operated via the DaVinci Xi system with a GelPoint Path. The mean estimated intraoperative blood loss was 88 mL and circular stapling was used to restore continuity in 80% of study patients. The overall postoperative complication rate was 35%, including one pelvic abscess, and the mean distal margin length was 3.1 ± 1.3 cm. They reported that all patients had complete or near complete mesorectal resections and three patients had CRM involved by cancer cells (≤1 mm) [45].

Ye et al. reported 13 cases of RTaTME in patients with rectal cancer demonstrating the feasibility of this innovative approach. The median docking time was 18 min, median transanal phase time was 95 min, and median total operation time was 240 min. Median estimated blood loss was 60 mL, the median number of lymph nodes retrieved was 15 and median length of postoperative hospital stay was 7 days, without mortality recorded. Three postoperative complications including one anastomotic leak and one prolonged ileus were reported, with no requirement for further intervention. Patients were followed up for a median of 15 months, and no local tumor recurrences, metastasis or deaths were reported [46].

The results of these experiences are summarized in Table 1.

### Emerging Robotic Systems

Despite the well described benefits, the presence of external arm clashes and internal conflicts make the multi-arm robot inappropriate for single port surgery.

The introduction of the robotic platform based on single-port access and the implementation of smaller, more flexible robotic systems designed for true natural orifice procedures, may represent the start of a new era for robot-assisted transanal surgery (Table 2).

In a correspondence article, Samalavicius et al. reported on a 57-year-old patient with an ulcerated rectal tumour 5 cm from the anal verge who underwent robotic-assisted TaTME using a Senhance Transenterix robotic system. After failure of long-course chemoradiotherapy and a watch-and-wait strategy, the patient underwent an uneventful procedure, demonstrating that RTaTME using the Senhance robotic system is a good and feasible option for low-lying rectal cancer [47].

Atallah et al. demonstrated in a cadaveric model the preclinical feasibility of the Versius surgical modular robotic system for taTME. Using this modular robotic system, one surgeon performed the abdominal portion of the operation, including colonic mobilization and vascular pedicle ligation, while simultaneously a second surgeon performed the transanal portion of the operation to the point of rendezvous at the peritoneal refection, where the operation was completed cooperatively. The operation was successfully completed in 195 min demonstrating the theoretical advantage of reducing surgical time and thereby reducing overall operative costs [48].

In 2017 the United States Food and Drug Administration approved the Flex^®^ Robotic System and Flex^®^ Colorectal (CR) Drive (MedRobotics, Corp. Raynham, MA, USA), a semi-robotic apparatus for colorectal surgery specifically indicated for transanal endoluminal applications, as well as for more radical resection. Atallah et al. used the flexible robotic system to perform taTME, showing its feasibility and potentiality to perform operative tasks not otherwise possible with conventional methods [49].

Similarly, taTME was performed by two surgeons in six fresh human cadaveric specimens using the same platform, with or without transabdominal laparoscopic assistance, simulating both mid- and low-rectal resections [50].

In a cadaveric study, the da Vinci^®^ SP Surgical System was shown to be a realistic platform for the future of endoluminal surgery [51] and in 2020 the first clinical experience performing a single-port left colectomy using the SP robot (SPr SILS left colectomy) was described [52]. In a subsequent feasibility study, Dr. Marks expanded the use of the SP robot performing TaTME, including transanal splenic flexure release and high ligation of the IMA, but the results of these experiences have not yet been published.

Recently Ribero et al. published a preclinical study to establish the technical feasibility of RtaTME using the da Vinci^®^ SP, simulating two clinical scenarios of rectal cancer at different heights from the anal verge (at 1 cm and at >4 cm respectively), in two fresh cadavers. The GelPOINT Path was used as transanal access platform through which the 2.5 cm single port robotic trocar and a 10 mm sleeve were inserted. A complete taTME was performed with an intact mesorectal fascia in both cases. Operation times were 124 and 106 min with about 15 min of non-console time for robot positioning and docking [53].

In a preclinical study in a male human cadaver the da Vinci^®^ SP Surgical System was employed to realize the transanal and abdominal parts of the taTME procedure, in a sequential fashion with a one-team approach. This experience demonstrated the technical feasibility of a dual field procedure, with operative time of 189 min for the perineal phase and 43 min for the abdominal procedure, and good quality of resected specimen [54].

With these advances, the SP robot demonstrates significant surgical milestones in the field of transanal and other natural orifice surgery.

## 4. Benefits and Limitations

### 4.1. Technical Advantages

Compared to conventional laparoscopy, current robotic platforms provide a stable camera with a magnified 3DHD vision delivering an immersive surgical experience, with true depth perception which allows the clear identification of tissue planes and anatomical structures.

Modelled after the human wrist, the EndoWrist^®^ instruments offer a greater range of motion than the human hand, providing maximum responsiveness and allowing rapid and precise suturing, dissection and tissue manipulation [20].

These technical advantages, more relevant in such a restricted surgical field, are expected to allow a fine TME with preservation of the integrity of the fascia and consequent optimal oncological results. On the other hand, the better identification and preservation of autonomic nerves should result in improved functional outcomes, with reduced sexual dysfunction, anterior resection syndrome or urinary retention.

Finally, but no less important, robotics offers optimal ergonomics for operating surgeons, with reduced musculoskeletal discomfort, and this could reflect a better quality of surgery, especially for such challenging technical operations.

### 4.2. Technical Limitations

The loss of force feedback, coupled with the inherent ability of robotic surgical systems to apply strong compressive and shear forces, have led to an increased risk of excessive tissue trauma, representing the major technical limitation of the available robotic platforms [55,56].

With the aims of imitating natural touch and improving the effectiveness of haptic feedback in tissue grasping and manipulation, in a benchmark study by Abiri et al. a multi-modal pneumatic feedback system, designed to allow for tactile, kinesthetic and vibrotactile feedback, was mounted on a da Vinci^®^ Surgical System, demonstrating the possibility of achieving average grip forces closer to those normally possible with the human hand [57].

With the advancement of technology, it appears mandatory that haptic feedback modalities are developed, becoming a standard feature of commercially available surgical robots.

As a consequence, new-generation robotic platforms such as the Senhance Surgical Robotic System and the REVO-I Robot Platform incorporate haptic feedback systems [58].

Moreover and more importantly, da Vinci^®^ robotic arms are large and intrusive, making it very challenging to work in the setting of single-port or transanal surgery, because of external clashes and collisions. At this stage, current experiences of robotic-assisted transanal surgery have demonstrated the feasibility of the approach, but have highlighted the limitations of the available robotic systems, relying on the potential benefits of emerging flexible or miniaturized systems.

### 4.3. Costs

Despite the efforts aimed at containing increasing expenditure, overall worldwide health care spending remains on an unsustainable course. As a consequence, in consideration of limited health resources, a relevant focus has been placed on the assessment of the economic impact of robotics, given that the attributed increase in costs, associated with equivocal evidence of improved clinical outcomes, is the most significant barrier preventing the diffusion of this technology.

It is the case that da Vinci^®^’s supremacy in the market of robotic surgery, in the absence of real competition, whilst producing a potential stagnation in the advancement of technology, has mostly led to a growth in costs. On the other hand, the accurate assessment of the financial impact of robotic surgery is a challenging operation, as total costs include direct, indirect and intangible costs.

Direct costs can be fixed costs (to buy and maintain the robotic system), and variable costs (consumable instruments), but to better understand whether robotic surgery is beneficial compared to other available techniques, it is mandatory to also capture indirect costs (loss of productivity, trips to hospital) and out-of-pocket costs borne by patients after discharge.

No economic data are available concerning RTaTME but almost all studies on robotic TME showed higher costs compared with laparoscopic TME, with similar overall clinical outcomes. After a learning curve for robotic TME, the operative costs could be reduced, but the total costs including fixed costs would still be higher because of the expensive purchasing charge for the robotic system (average cost of a robotic platform is $1–$2.3 million) [59].

The ROLARR trial showed that health-care costs in the robotic-assisted laparoscopic group (£11,853 or $13,668) were higher than in the conventional laparoscopic group (£10,874 or $12,556), because of a longer mean use of the operating theatre and the mean cost of instruments [60].

Baek et al. in a cost analysis from a single institute in South Korea reported that operative charges were significantly higher in the robotic surgery group (8849 vs. 2289 USD, *p* ≤ 0.001), while the charge for anesthesia, laboratory, radiology, nursing care and medical therapy was not different between two groups [61].

On the other hand, Kim et al., in a propensity score-matching analysis of cost-effectiveness of robotic versus laparoscopic surgery, showed similar short-term clinical outcomes, but higher costs in all categories of charges for the robotic group (total hospital charges, patients’ payment, operative charges, anesthetic charges, and postoperative management charges) [62].

Ramji et al., comparing the clinical and economic outcomes between open, laparoscopic and robotic approaches to rectal cancer surgery, did not find any difference for total and operative costs between the open and laparoscopic method, whereas the median costs of each robotic operation increased approximately by 6000 CAD [63].

In the experience of Ielpo et al. comparing clinical outcomes and costs of robotic versus laparoscopic surgery for rectal cancer, the mean overall costs were similar in both groups (7279.31 vs. 6879.80, EUR, *p* = 0.44), but fixed costs for robotic surgery were not included, thus minimizing the real financial impact of robotic devices on health spending [64].

Morelli et al. demonstrated that total costs (12,283.5 vs. 7619.8 EUR, *p* < 0.001), and variable costs (10,614.6 vs. 7585.4 EUR, *p* < 0.001) were higher in the robotic TME group compared with the laparoscopic TME group, as well as within the robotic group itself in the first phase of the learning curve, reflecting how the advancement of robotic experience produced a reduction of operative time and consequently of overall costs. Excluding fixed costs, the variable operative costs were similar between the robotic group, for the achievement of proficiency, and the laparoscopic TME group (*p* = 0.084) [65].

This demonstrates how relevant is robotic expertise to the potential reduction of indirect costs, alongside institutional case volume, standardization of procedures and efficiency of surgical teams.

Despite that analysis of indirect costs is not available in the literature, it is conceivable that the described potential advantages of robotic surgery in terms of reduction of conversion rate and hospital stay may translate into lower expenditure.

A recent retrospective, propensity score-weighed analysis using insurance claims from the MarketScan database showed that the robotic approach was associated with lower out-of-pocket costs for five types of common oncological procedures compared to open surgery [66].

The analysis of indirect costs and the evaluation of quality of life measures, including sexual, urinary and bowel functions, require further research to accurately compare the outcomes of the different surgical alternatives.

So far, results from the available literature suggest that robotic-assisted surgery for rectal cancer is unlikely to be cost-saving, mainly considering the cost of purchase and maintenance of the system.

In the near future, positive competition among companies will foster the introduction of new robotic systems and the expected gradual decrease of the platform price will make it cost-effective, with the same clinical outcomes as alternative operation techniques.

## 5. Learning Curve of RTaTME

For such a challenging surgical procedure, one of the main areas of research is to identify the phases of the learning curve and to establish appropriate training, in order to guarantee patients’ safety and to provide inexpert surgeons with adequate proctorship.

Despite the fact that robotic surgery is generally more intuitive and easier to learn than laparoscopic surgery, a learning curve is unavoidable and special training programs for the development of new surgical skills appear mandatory. FDA enforced companies responsible for robotic systems to develop dedicated, structured educational programs for surgeons and an official certification based on a formal curriculum for skills and procedures has been recommended also by The European Association of Endoscopic Surgeons (EAES).

Supporters of the TaTME technique have highlighted the potential benefits, mainly in selected high-risk patients, without ignoring that the new bottom-up approach is associated with a significant learning curve which, from the evaluation of major post-operative complications, is estimated at 40–50 cases [67].

On the other hand, robotic transabdominal TME should include at least 20–23 cases to gain proficiency, which is in any case faster than for laparoscopy [68,69].

Being still in its infancy, no formal assessment of the learning curve for RTaTME is reported in the literature, but it is intuitive that the steep learning curve of so complex a technique would probably be favoured by robotic technology.

“Operative time”, “bleeding” or “conversion” have been evaluated as markers of expertise in most of the publications [69,70,71], but the most critical variable to be assessed in rectal cancer surgery is the difference between learning and competent surgeons in terms of the quality of the resected specimen [72].

If robotics does not modify the operative steps and the intrinsic complexity of this procedure, it may ease or simplify its technical aspects, making minimally invasive surgery feasible by less experienced surgeons in more patients, thus shortening the time needed for the achievement of proficiency.

On the other hand, limitations imposed by the currently available robotic platforms may at this stage counterbalance the advantages, making the learning curve steeper than expected.

## 6. Conclusions

Robotic-assisted surgery was introduced at the beginning of the new millennium, showing obvious advantages over laparoscopy in terms of visualization, manipulation and ergonomics.

With proper training, TaTME can be considered a real game-changer in the surgical management of rectal cancer. The accuracy of transanal dissection may be improved by the use of robotic assistance, which expresses its potentialities even better in confined spaces such as the pelvic outlet.

To date only a few experiences have demonstrated the feasibility and safety of this approach, with oncological results expected to be not inferior compared to conventional TaTME.

Increased costs, poor availability and dedicated training are still relevant barriers which prevent the wide adoption of this system, also because, at this stage, no important benefits have been demonstrated yet for robotics compared with the other available surgical alternatives.

In the near future, emerging robotic platforms will lead to major competition and consequent reduction of costs, while the development of miniaturization of the surgical instrumentation will start a new era for endoluminal surgery.

These advancements, associated with technical refinements and standardized training programmes, may allow robotic surgery to become the gold standard for TaTME.

## Figures and Tables

**Table 1:** Summary of published experience of RTaTME performed with da Vinci^®^ Robotic platform.

	Atallah (2013)	Atallah (2014)	Verheijen (2014)	Huscher (2015)	Gomez-Ruiz (2015)	Kuo (2016)	Monsellato (2019)	Hu (2020)	Ye (2020)
Number of patients	1	3	1	7	5	15	3	20	13
Abdominal approach	Laparoscopic	Laparoscopic	Laparoscopic	Laparoscopic	Robotic	Single port robotic + assistant port	Robotic 2, laparoscopic 1	Laparoscopic	Robotic 9, Laparoscopic 4
Transanal platform	GelPoint Path (daVinci^®^ Si)	GelPoint Path (daVinci^®^ Si)	GelPoint Path (daVinci^®^ Si)	GelPoint Path (daVinci^®^ Si)	PAT * + GelPoint Path (daVinci^®^ Si)	GelPoint Path (daVinci^®^ Si)	GelPoint Path (daVinci^®^ Si)	GelPoint Path (daVinci^®^ Xi)	GelPoint Path (daVinci^®^ Si)
Two-team approach	No	No	No	No	No	No	1/3	20/20	4/13
BL (mL)	140	200	50	n/a	90 (25–120)	33 (30–50)	n/a	82 (30–500)	60 (50–100)
LOS (days)	No	4.3	3		6 (5–7)	12.2 (10–14)	10 (7–15)	8.8 (6–24)	7 (6–10)
Conversion	No	No	No	No	No	2/15	No	No	No
Hand-sewn anastomosis	0/1	2/3	0/1	0/7	2/5	15/15	3/3	2/20	8/13
Defunctioning stoma	Terminal ileostomy	Yes	Yes	Yes	Yes	5/15	Yes	14/18	Yes
Operative time (min)	381	376	205	165.7 (85–220)	398 (270–450)	473 (335–569)	550 (440–600)	172.3 (135–215)	240 (195–270)
Complications	No	1 Pulmonary embolism 1 Peristomal dermatitis/ dehydration	No	1 anastomotic bleeding	1 anastomotic leak	1 mechanical bowel obstruction, 1 wound infection	1 acute renal failure	7/20 (no anastomotic leaks reported)	1 post-op ileus 1 duodenal hemorrage 1 anastomotic leakage
TME quality C/NC/I	0/1/0	1/2/0	1/0/0	6/1/0	5/0/0	15/0/0	3/0/0	18/2/0	8/5/0
CRM involvement	No	No	No	No	No	No	No	3/20	No
Distal margin involvement	No	No	No	No	No	No	No	No	No

BL: Blood loss; LOS: Length of hospital stay, C: Complete; NC: Near complete; I: Incomplete; * PAT (‘Puerto Acceso Transanal’—Developia-HUMV, Santander, Spain).

**Table 2:** Summary of published experience of RTaTME performed with emerging robotic platforms.

	Samalavicius (2020)	Atallah (2019)	Carmicheal (2019)	Ribeiro (2021)	Kneist (2020)
Number of cases	1 patient	1 fresh human cadaver	6 fresh human cadavers	2 fresh cadavers	1 fresh human cadaver
Robotic platform	Senhance Transenterix	Versius	Flex^®^ System	daVinci^®^ SP	daVinci^®^ SP
Abdominal approach	Robotic	Robotic with Versius	n/a	n/a	Robotic with daVinci^®^ SP
Transanal platform	n/a	GelPoint Path	n/a	GelPoint Path	GelPoint Path
Two-team approach	n/a	Yes	n/a	n/a	No
Hand-sewn anastomosis	Yes	n/a	n/a	n/a	n/a
Operative time (min)	n/a	195	n/a	n/a	232
Complications	No	n/a	n/a	n/a	n/a
TME quality	n/a	Near complete	Complete 4 Incomplete 2	Complete 3	Good
CRM involvement	No	n/a	n/a	n/a	n/a
Distal margin involvement	No	n/a	n/a	n/a	n/a
